# Cell Shock Absorption via Stress Relaxation Hydrogel Microspheres for Alleviating Endoplasmic Reticulum Stress in Chondrocytes

**DOI:** 10.34133/research.0777

**Published:** 2025-07-17

**Authors:** Ding Zhao, Yihan Li, Lei Xiang, Qimanguli Saiding, Zhiqi Lin, Zhengwei Cai, Juan Wang, Wenguo Cui

**Affiliations:** Department of Orthopaedics, Shanghai Key Laboratory for Prevention and Treatment of Bone and Joint Diseases, Shanghai Institute of Traumatology and Orthopaedics, Ruijin Hospital, Shanghai Jiao Tong University School of Medicine, Shanghai 200025, P. R. China.

## Abstract

Chronic mechanical vibrations and endoplasmic reticulum (ER) stress are major contributors to osteoarthritis (OA) progression. This study proposes a novel “cellular shock absorption” approach by developing viscoelastic hydrogel microspheres with tunable stress relaxation properties. By modulating the chemical bonds in the hydrogel network through oxidation and hydrazine coupling reaction, hydrogel microspheres capable of absorbing shock and reducing mechanical stimulus-induced ER stress in chondrocytes are created. Cationic liposomes, modified with the cartilage-targeting peptide Wyrgrl and loaded with tauroursodeoxycholic acid (TUDCA), are encapsulated within these hydrogel microspheres. The microspheres not only dissipate intra-articular impact forces, reducing vibration and pressure transmission, but also provide sustained release of TUDCA, alleviating ER stress and slowing OA progression. In vitro studies showed that the stress relaxation time constant (τ) of the microspheres was tuned to 23.81 s, closely resembling the mechanical properties of the cartilage matrix. This property, combined with targeted TUDCA delivery, reduced Grp78 and CHOP expression, alleviating ER stress and inhibiting chondrocyte apoptosis. In vivo, the microspheres preserved joint cartilage structure, suppressed ER stress responses, and substantially delayed OA progression. This strategy presents a promising approach to mitigating cartilage damage and delaying OA by reducing mechanical stress and alleviating ER stress.

## Introduction

Cellular motion underpins the functionality of living systems. Cells interact with their mechanical environment through mechanotransduction and substrate-dependent movement [1–3]. Different intensities of mechanical stress induce distinct cell motility patterns. Moderate mechanical stress promotes directional movement and distribution, enhancing migration efficiency and facilitating tissue repair [4,5]. In contrast, excessive mechanical stress activates endoplasmic reticulum (ER) stress, oxidative stress, and key signaling pathways [e.g., p38 mitogen-activated protein kinase (MAPK), c-Jun N-terminal kinase (JNK), and nuclear factor κB (NF-κB)], suppressing cell migration and leading to apoptosis or necrosis [6,7]. Moreover, cellular motion reciprocally influences the mechanical environment. For instance, during wound healing, fibroblasts migrate to injury sites, releasing matrix metalloproteinases (MMPs) to degrade extracellular matrix (ECM) and enhance migration [8]. However, excessive fibroblast activity can cause pathological changes, such as aberrant tissue remodeling during liver or lung repair, resulting in fibrotic diseases. In osteoarthritis (OA), the heightened sensitivity of chondrocytes to mechanical stress renders cartilage vulnerable to mechanical damage [9]. Excessive mechanical stress induces aberrant chondrocyte behavior, disrupting normal function, accelerating apoptosis, and exacerbating ECM degradation, ultimately driving cartilage degeneration and OA progression [10–12]. Maintaining cellular motion within a stable mechanical environment is therefore critical for tissue repair and functional homeostasis [13,14].

Current OA treatments, such as hyaluronic acid (HA) injections, alleviate symptoms by lubricating joints and facilitating smoother tissue movement. Hydrogel microspheres, as a drug carrier, possess injectability and sustained-release properties, which can achieve precise drug delivery and prolonged release [15]. The structural parameters of microspheres, such as particle size and porosity, have a significant impact on cartilage regeneration [16]. Particle size affects the drug release rate and cell uptake efficiency, while porosity influences cell growth and the exchange of nutrients [17]. However, these approaches fail to address the fundamental mechanism of cartilage degradation—mitigating the aberrant mechanosensitivity and maladaptive interactions of chondrocytes in high-stress environments [18,19]. To counteract mechanical stress-induced cellular damage, novel therapeutic strategies are urgently needed to dampen the transmission of mechanical vibrations and pressures, thus reducing chondrocyte mechanosensitivity [20]. Such an approach could provide a “cellular shock-absorbing” effect to slow OA progression. While hydrogel microspheres have shown promise in cushioning cells [21], their inherent elasticity in the joint’s mechanical environment can inflict additional stress on cartilage [22]. Hence, developing stress-relaxing hydrogel microspheres that mimic the viscoelastic properties of native cartilage tissue is essential for attenuating mechanical vibrations, maintaining cellular motility, and preserving cell function [23].

Emerging evidence identifies the ER as a critical organelle for sensing mechanical signals, including stress, tension, and matrix stiffness [24]. The ER spans the cytoplasm and rapidly adapts its structure during cell deformation, interacting with cytoskeletal components such as actin and microtubules to mediate force transmission [25,26]. The actin-binding protein filamin A has been shown to interact with PERK, a key ER kinase, and IRE1, a major sensor of unfolded protein response (UPR) [27]. Beyond its role in stress responses, the ER is pivotal for synthesizing and folding ECM proteins in chondrocytes [28]. Excessive mechanical stress activates ER stress (ERS), leading to the accumulation of unfolded or misfolded proteins and triggering UPR through 3 primary sensors: IRE1, ATF6, and PERK. While UPR aims to restore ER homeostasis, prolonged activation drives chondrocyte apoptosis, exacerbating mechanical stress-induced damage [29,30]. Thus, therapeutic strategies targeting mechanical stress mitigation and ERS alleviation hold promise for effective OA treatment.

Herein, we propose a novel “cellular shock-absorbing hydrogel microsphere” strategy. Oxidized HA and hydrazine coupling reaction were employed to synthesize aldehyde-modified HAMA (HAMA-CHO) and amino-modified HAMA (HAMA-NH₂) [31,32]. Cationic liposomes loaded with TUDCA and Wyrgrl peptide (Lipo-Wyrgrl@TUDCA) were prepared via thin-film dispersion and subsequently incorporated into stress-relaxed HAMA microspheres using imine bonding and microfluidics. This approach yielded viscoelastic hydrogel microspheres (Stress-relaxed HAMA@Lip) with tunable stress relaxation properties [33,34]. These microspheres mitigate mechanical stress on cartilage and alleviate ERS-induced chondrocyte apoptosis, thereby decelerating OA progression. Stress-relaxed HAMA@Lip recapitulates the viscoelasticity of native cartilage, exhibiting enhanced molecular chain mobility and deformation under mechanical forces. Furthermore, TUDCA-loaded Lipo-Wyrgrl@TUDCA anchors to chondrocytes through electrostatic and Wyrgrl peptide-mediated targeting [35], aiding unfolded protein processing and alleviating ERS, thereby reducing chondrocyte apoptosis [36]. Stress relaxation properties and ERS modulation were evaluated in vitro using rheometry, immunofluorescence, quantitative polymerase chain reaction (qPCR), and Western blotting. In vivo, local injection of Stress-relaxed HAMA@Lip into a rat post-traumatic OA model significantly protected cartilage structure by suppressing ERS, as evidenced by micro-CT (computed tomography), immunohistochemistry, and immunofluorescence analyses. In summary, stress-relaxed HAMA@Lip effectively mitigates chondrocyte mechanical stress damage and ERS response, demonstrating potential to slow OA progression. Developing therapeutic strategies that attenuate excessive mechanical stress, restore cellular motility, and preserve cell function holds significant clinical relevance and offers new insights into treating diseases involving cell–mechanical environment interactions, such as muscle atrophy and cardiovascular disorders.

## Results

### Preparation and characterization of targeted chondrocyte liposomes for alleviating ERS

**Fig. 1.**
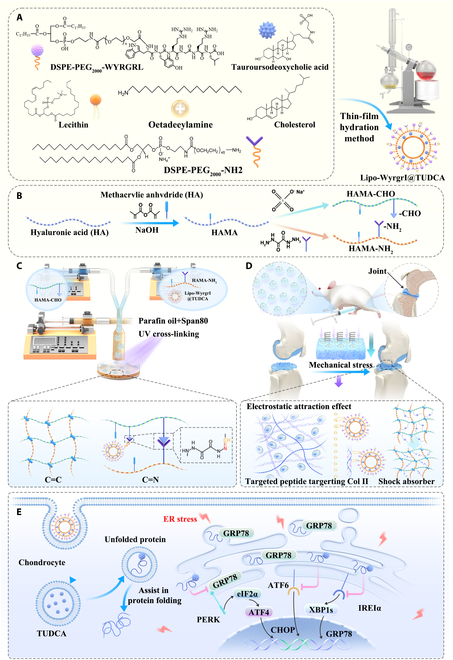
Schematic design and experimental flow diagram. (A) Preparation of cationic liposomes targeting cartilage. (B) Chemical modification of MA-grafted HAMA by oxidation and OPA/N-nucleophile condensation reactions. (C) Fabrication of stress-relaxed HAMA@Lip by multiphase microfluidics and UV crosslinking. (D) Targeting approach of stress-relaxed HAMA@Lip and mechanism of mechanical stress relaxation effect. (E) TUDCA alleviates apoptosis by aiding protein folding and inhibiting UPR.

Cationic liposomes are widely used as carriers for delivery of small molecule drugs [37,38]. To achieve targeted delivery of TUDCA to cartilage, we developed cationic liposomes specifically targeting cartilage tissue (Fig. 1). DSPE (1,2-distearoyl-sn-glycero-3-phosphoethanolamine)-PEG_2000_ (polyethylene glycol with an average molecular weight of 2,000)-Wyrgrl was synthesized by conjugating DSPE-PEG_2000_-NHS (N-hydroxysuccinimide) with the Wyrgrl peptide in a triethylamine environment, and the final product was obtained via dialysis and lyophilization. The successful synthesis of DSPE-PEG_2000_-Wyrgrl was confirmed by HPLC (high-performance liquid chromatography; Fig. S1), mass spectrometry (Fig. S2), and proton nuclear magnetic resonance spectroscopy (^1^H NMR; Fig. S3).

**Fig. 2.**
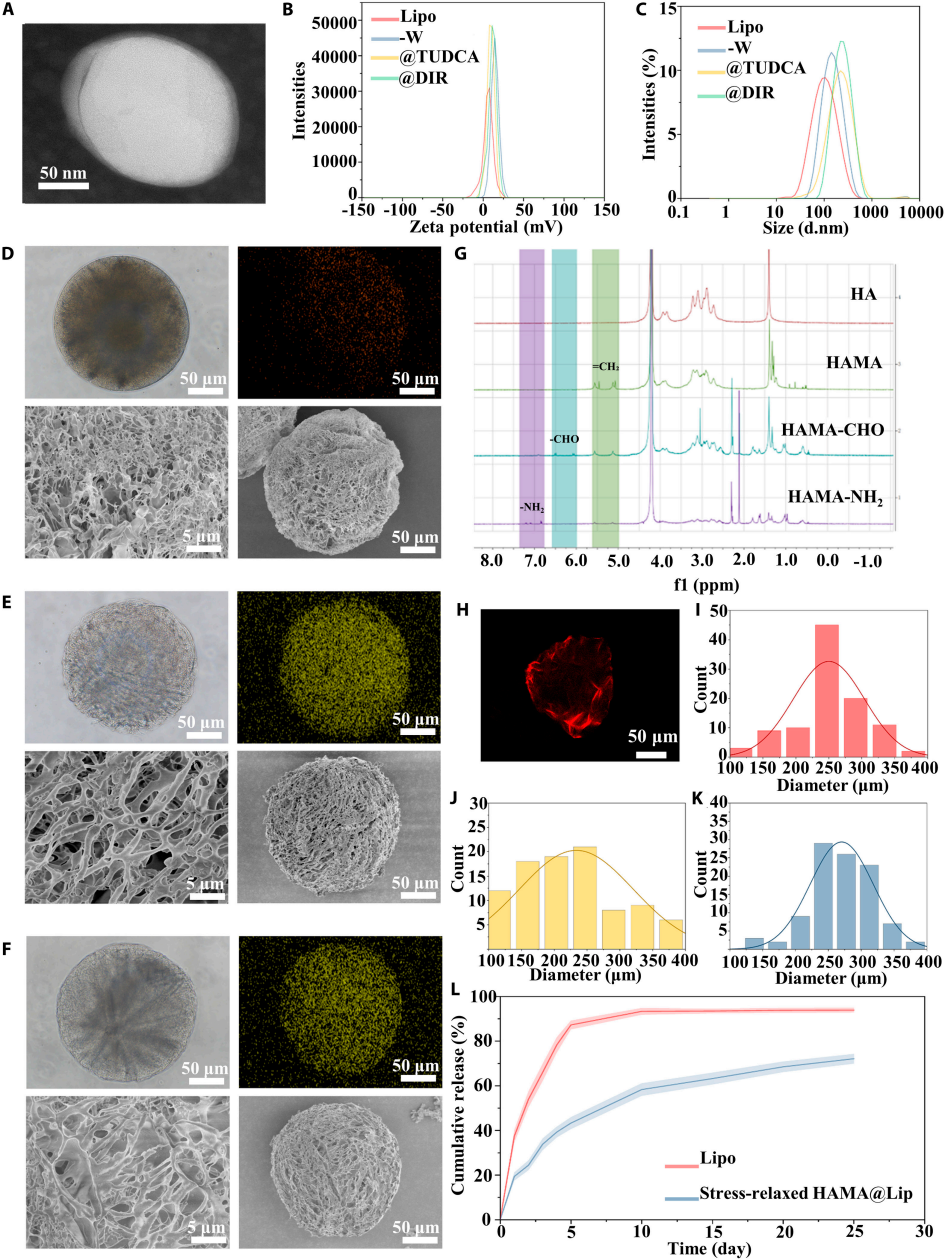
Construction and characterization of cationic liposomes and stress-relaxed HAMA@Lip. (A) TEM image of liposomes. (B and C) Particle size and zeta potential analysis of various liposome formulations, including unloaded liposomes (Lipo), WYRGRL-modified liposomes (Lipo-WYRGRL), TUDCA-loaded liposomes (Lipo-WYRGRL@TUDCA), and DiR-labeled liposomes (Lipo-WYRGRL@DiR), as determined by DLS. (D to F) Optical microscopy, phosphorus elemental mapping analysis, and SEM images of microspheres from different groups (HAMA microspheres, stress-relaxed HAMA, and stress-relaxed HAMA@Lip). (G) Proton nuclear magnetic resonance (^1^H NMR) spectra and substitution rates of various chemically modified HA products (HAMA, HAMA-CHO, and HAMA-NH₂). (H) Confocal microscopy images showing uniform loading of Lipo-WYRGRL@DiR on stress-relaxed HAMA. (I to K) Particle size distribution of microspheres from different groups (HAMA microspheres, stress-relaxed HAMA, and stress-relaxed HAMA@Lip). (L) Cumulative drug release profiles of Lipo-WYRGRL@TUDCA and stress-relaxed HAMA@Lip.

Lipo-Wyrgrl@TUDCA was then synthesized using DSPE-PEG_2000_-NH_2_, DSPE-PEG_2000_-Wyrgrl, phosphatidylcholine, cholesterol, stearylamine, and TUDCA. The encapsulation efficiency of TUDCA, as measured by ultraviolet–visible (UV–Vis) spectrophotometry, was approximately 84.65% (Fig. S5). TEM (transmission electron microscopy) images showed that Lipo-Wyrgrl@TUDCA exhibited a multilayered structure with a particle size around 150 nm (Fig. 2A).

To further investigate liposome loading within hydrogel microspheres, we introduced DIR (1,1′-dioctadecyl-3,3,3′,3′-tetramethylindotricarbocyanine iodide)-labeled liposomes and synthesized 4 types of liposomes: Lipo, Lipo-Wyrgrl, Lipo-Wyrgrl@TUDCA, and Lipo-Wyrgrl@DIR. Dynamic light scattering (DLS) was used to analyze the particle size and zeta potential of these liposomes. The inclusion of positively charged stearylamine in all formulations resulted in a similar zeta potential of approximately +12 mV for all liposomes, reflecting their cationic characteristics (Fig. 2B). Particle size analysis revealed that the diameter of Lipo was approximately 90 nm, Lipo-Wyrgrl was around 110 nm, and the modified Lipo-Wyrgrl@TUDCA and Lipo-Wyrgrl@DIR were both about 160 nm, exhibiting a polydispersity index (PDI) of 0.24, indicating good stability (Fig. 2C).

The dimensions and surface charge of liposomes play a crucial role in determining their functionality. The positive charge facilitates electrostatic adsorption, overcoming the charge barrier and enabling efficient localization to cartilage tissue [39]. Furthermore, the size of these liposomes, combined with Wyrgrl peptide modification, allows them to penetrate the cartilage matrix, target chondrocytes, and achieve sustained release of TUDCA.

### Preparation and characterization of hydrogel microspheres mimicking cartilage matrix for shock absorption

HA (molecular weight 168 kDa) was chemically modified to investigate the effects of material composition on modulus and viscoelastic properties [40]. HA was first reacted with methacrylic anhydride (MA) under ice-bath conditions overnight to introduce photo-crosslinking functionality, yielding HAMA (methacrylate hyaluronic acid) [41]. The synthesis of HAMA was confirmed through ^1^H NMR, showing a degree of substitution of approximately 31% (Fig. 2G). Next, HAMA was oxidized with sodium periodate to produce aldehyde-modified HAMA (HAMA-CHO), with NMR analysis confirming a substitution degree of ~27% (Fig. 2G). Amino-modified HAMA (HAMA-NH_2_) was synthesized by reacting HAMA with hydrazine dihydrochloride, achieving a substitution degree of ~28.67% as verified by NMR (Fig. 2G).

The Schiff base reaction between aldehyde and amino groups forms imine bonds (C=N), with bond energies of 100 to 150 kJ/mol, weaker than typical covalent bonds (300 to 400 kJ/mol) [42]. This weaker crosslinking structure dissociates under stress or strain, allowing the hydrogel matrix to flow. By adjusting the material ratios, the initial swelling and degradability of the matrix can be tuned to regulate stress relaxation properties, mimicking the viscoelasticity of the cartilage ECM and mitigating elastic stress damage to knee cartilage.

The synthesized materials (HAMA, HAMA-CHO, HAMA-NH_2_) were each dissolved in deionized water at 2% concentration with 0.5% photoinitiator [6-methoxy-2-naphthaldehyde (NAP)] [43]. After dissolution, blue light crosslinking was performed to assess gel formation (Fig. S4). Experimental groups were classified as follows: (a) HAMA group; (b) HAMA:HAMA-CHO (1:1) group; (c) HAMA:HAMA-NH_2_ (1:1) group; (d) HAMA-NH_2_:HAMA-CHO (1:1) group; (e) HAMA-CHO:HAMA-NH_2_:Lipo-Wyrgrl@TUDCA (ratios 1:1:1, 1:2:1, 1:4:1, 2:1:1, 4:1:1, 2:4:1, 4:2:1, and 1:1:2) groups.

**Fig. 3.**
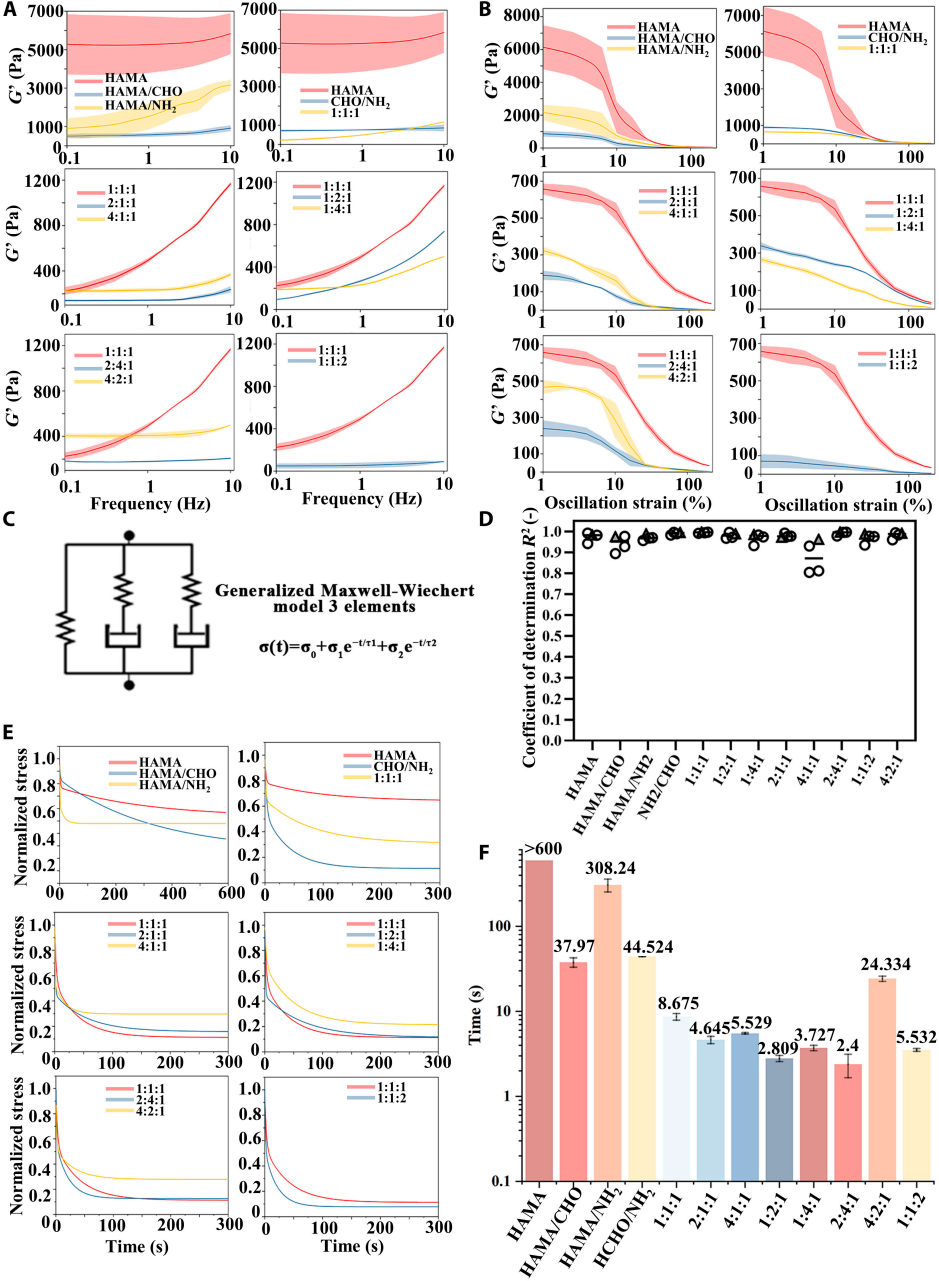
Mechanical characterization of stress-relaxed hydrogels with varying compositions. (A) Frequency sweep (*n* = 3) curves of hydrogels with different compositions at 37 °C under a fixed strain of 0.1% over a frequency range of 0.1 to 10 Hz; shaded areas represent the standard deviation within each group. (B) Amplitude sweep (*n* = 3) curves of hydrogels with different compositions at 37 °C under a fixed frequency of 1 Hz over a strain range of 1% to 100%; shaded areas represent the standard deviation within each group. (C) Experimental data fitted using the generalized 3-element Maxwell–Wiechert model. (D) The Maxwell–Wiechert model accurately fits the experimental data, with the goodness of fit determined by the coefficient of determination (*R*^2^). Hollow circles represent individual data points for each group (*n* = 3), and gray triangles indicate the mean values for each dataset. (E) Stress relaxation fitting curves of hydrogels with different compositions at 37 °C. The fitted data for each group were derived from independently prepared hydrogels (*n* = 3). (F) Stress relaxation constants of hydrogels with different compositions (*n* = 3) over an observation period of 600 s. Error bars are not shown for the HAMA group as the relaxation times exceeded 600 s.

Each group included 0.5% NAP as the photoinitiator. Rheological analysis of mechanical properties was performed using a rheometer (Fig. 3A), including frequency (5% strain) and amplitude sweeps (1 Hz) (Fig. 3A and B). The storage modulus of photo-crosslinked HAMA alone was ~5,000 Pa. However, incorporating HAMA-CHO and HAMA-NH_2_ at a 1:1 ratio reduced the storage modulus to ~600 Pa, demonstrating the impact of imine bonds on material stiffness. Adding Lipo-Wyrgrl@TUDCA to the 1:1 mixture further reduced the modulus to ~400 Pa due to the inability of liposomes to form strong covalent bonds. The variations in storage modulus among the different groups reflect differences in the bonding strength within the material. When the reaction between aldehyde groups and amino groups reaches the optimal stoichiometric ratio, the crosslinking density of imine bonds is maximized, resulting in the highest storage modulus. This explains why the 1:1 group exhibits the maximum modulus. In contrast, cationic liposomes form fewer effective bonds with the material. Therefore, increasing the proportion of liposomes reduces the storage modulus.

Viscoelasticity was characterized using stress relaxation testing, where a constant strain was applied, and stress was monitored over time to assess material resistance to deformation [44,45]. The stress relaxation time constant (τ), defined as the time for stress to relax to half its initial value (τ_1/2_), was fitted using a generalized Maxwell–Wiechert 3-element model (Fig. 3C). The fitted curves were well correlated with the experimental data, with *R*^2^ values ranging from 0.95 to 0.99 across all tested groups (Fig. 3D), confirming the robustness and reliability of the viscoelastic model fitting. Healthy cartilage tissue has a τ of ~20 s [11,45–48]. Among the tested formulations, the group with a HAMA-CHO:HAMA-NH_2_:Lipo-Wyrgrl@TUDCA ratio of 4:2:1 exhibited a τ of 23.81 s (Fig. 3F), closely mimicking healthy cartilage viscoelasticity. This group, with a modulus of ~400 Pa, demonstrated compatibility with joint surface irregularities and movement-induced deformation, effectively absorbing mechanical shock and reducing joint damage. This formulation was termed Stress-relaxed HAMA.

Stress-relaxed HAMA microspheres were prepared using a microfluidic device [49], producing uniformly dispersed microspheres with a diameter of 232.16 ± 88.98 μm under optical microscopy (Fig. 2I to K). The electrostatic dipole effect between liposomes and HA, along with imine bond formation between DSPE-PEG_2000_-NH_2_ in liposomes and HAMA-CHO, effectively anchored liposomes within the hydrogel matrix, enhancing system stability. DIR-labeled liposomes (replacing TUDCA) demonstrated uniform distribution and stability within microspheres under confocal microscopy (Fig. 2H).

The freeze-dried microspheres were examined by scanning electron microscopy (SEM) and energy-dispersive spectroscopy (EDS), revealing a decrease in porosity as component complexity increased: 41.16%, 31.40%, and 26.10% for HAMA, Stress-relaxed HAMA, and Stress-relaxed HAMA@Lipo-Wyrgrl@TUDCA microspheres, respectively (Fig. 2D to F). Elemental mapping of C, N, O, and P confirmed liposome distribution (Fig. S5). Drug encapsulation efficiency was 55.95% (Fig. S6), with Lipo-Wyrgrl@TUDCA releasing 80% of TUDCA by day 4.5 at 37 °C, while Stress-relaxed HAMA@Lipo-Wyrgrl@TUDCA sustained TUDCA release for 25 d, achieving a cumulative release of 72.15% by day 25 (Fig. 2L). In addition, the in vitro degradation profile of the stress-relaxed HAMA@Lipo-Wyrgrl@TUDCA microspheres showed a gradual mass loss, with only 11.05% of the original mass remaining after 10 d of enzymatic incubation in hyaluronidase-containing phosphate-buffered saline (PBS) at 37 °C (Fig. S7).

### Biocompatibility of Stress-relaxed HAMA@Lipo-Wyrgrl@TUDCA

To evaluate biocompatibility, Stress-relaxed HAMA, Lipo-Wyrgrl@TUDCA (hereafter referred to as Lip), and Stress-relaxed HAMA@Lip were cocultured with primary rat chondrocytes. Cell viability was assessed at 24, 48, and 72 h using live/dead cell staining with a calcein-AM/PI (propidium iodide) double staining kit, followed by a CCK-8 (Cell Counting Kit-8) assay.

**Fig. 4.**
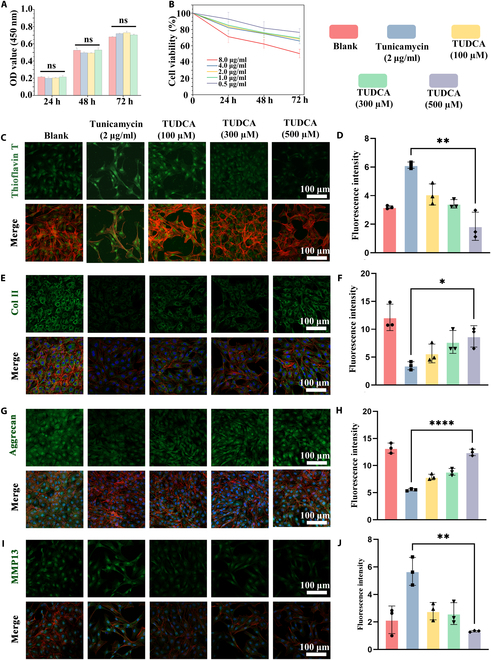
Stressed-relaxed HAMA@Lip exhibits good biocompatibility and alleviates chondrocyte ERS and OA phenotypes. (A) CCK-8 assay results for chondrocytes treated with blank, Lip, stress-relaxed HAMA, and stress-relaxed HAMA@Lip on days 1, 2, and 3 (Blank, Lipo-Wyrgrl@TUDCA; Stress-relaxed HAMA; Stress-relaxed HAMA@Lipo). (B) Cell growth curves under different concentrations of tunicamycin. (C and D) ThT staining results and corresponding fluorescence quantification for different treatment groups (*n* = 3). (E and F) Col II staining results and corresponding fluorescence quantification for different treatment groups (*n* = 3). (G and H) Aggrecan staining results and corresponding fluorescence quantification for different treatment groups (*n* = 3). (I and J) MMP13 staining results and corresponding fluorescence quantification for different treatment groups (*n* = 3). One-way ANOVA with Tukey’s post hoc test. ns: no significance, *P < 0.05, **P < 0.01.

The results demonstrated no significant changes in cell density or the proportion of dead cells among the different groups at any time point (Fig. S8). Furthermore, the absorbance values at 450 nm measured by the CCK-8 assay revealed no statistically significant differences between the groups (Fig. 4A), indicating that Stress-relaxed HAMA@Lipo-Wyrgrl@TUDCA exhibits good biocompatibility.

### Stress-relaxed HAMA@Lip alleviates ERS and OA phenotype in chondrocytes

The ER is a crucial organelle for protein synthesis in chondrocytes. Given the abundance of ECM in articular cartilage, chondrocytes must secrete large amounts of proteins to maintain matrix stability [50]. In OA, chondrocytes often exhibit ERS. Previous studies have demonstrated that the expression of ERS-related markers increases as OA progresses, leading to reduced ECM synthesis, decreased cartilage thickness, and fibrotic tissue formation.

To induce ERS in vitro, chondrocytes were treated with tunicamycin, a compound that inhibits glycosylation and leads to the accumulation of misfolded proteins in the ER [51,52]. A tunicamycin concentration of 2 μg/ml was chosen based on CCK-8 viability assays (Fig. 4B). TUDCA, a water-soluble bile acid that functions as a chemical chaperone, was used to alleviate ERS by assisting the refolding of misfolded proteins and preventing their aggregation [53,54]. Experiments were conducted using TUDCA at concentrations of 100, 300, and 500 μM.

Fluorescence microscopy using thioflavin T (ThT), a dye that binds to protein aggregates [55,56], revealed a significant increase in misfolded protein aggregates following tunicamycin treatment. TUDCA reduced these aggregates in a dose-dependent manner, with the 500 μM concentration lowering aggregates to levels below the untreated control group (Fig. 4C and D). Immunofluorescence staining showed that tunicamycin treatment reduced the expression of type II collagen (Col II) and aggrecan, key ECM components, while enhancing MMP13 expression. Treatment with Stress-relaxed HAMA@Lip restored Col II and aggrecan levels and significantly reduced MMP13 expression, indicating its potential to mitigate OA-related changes (Fig. 4E to J).

**Fig. 5.**
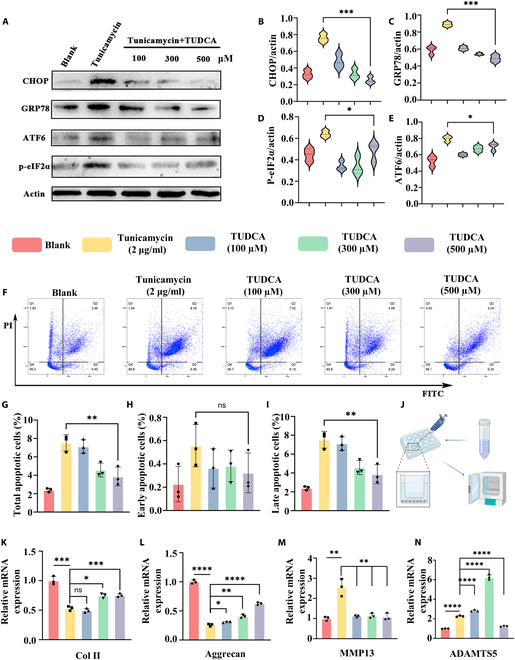
Stressed-relaxed HAMA@Lip promotes protein folding, alleviates ERS, and reduces apoptosis. (A) Western blot analysis of CHOP, GRP78, ATF6, p-eIF2α, and actin expression levels across different treatment groups. (B to E) Quantitative analysis of CHOP, GRP78, ATF6, and p-eIF2α expression levels normalized to actin (*n* = 3). (F) Flow cytometry analysis of early and late apoptosis in different treatment groups. (G to I) Quantitative statistics of total apoptotic cells, early apoptosis, and late apoptosis proportions in different treatment groups (*n* = 3). (J) Schematic illustration of coculture of Stressed-relaxed HAMA@Lip with chondrocytes. (K to N) Expression levels of OA-related genes in chondrocytes after coculture (*n* = 3). One-way ANOVA with Tukey’s post hoc test. ns: no significance, *P < 0.05, **P < 0.01, ***P < 0.001, **** *P* < 0.0001.

Western blot analysis of ERS markers showed that GRP78, a molecular chaperone assisting in protein folding, decreased with increasing TUDCA concentrations [57]. Similarly, TUDCA significantly reduced p-eIF2α and CHOP (C/EBP homologous protein) expression, markers associated with apoptosis induction. ATF6, a key factor in the UPR, was also down-regulated following TUDCA treatment [58] (Fig. 5A to E). Flow cytometry further confirmed that TUDCA effectively reduced late apoptosis in chondrocytes under ERS conditions, although its effect on early apoptosis was limited. In the model group treated with tunicamycin, the total proportion of apoptotic cells was 8.09% ± 0.91%, whereas in the control group, the overall apoptosis rate was 2.63% ± 0.30%. The group treated with the highest concentration of TUDCA exhibited a total apoptotic cell rate of 4.19% ± 1.18%. However, the proportion of early apoptotic cells did not differ significantly among the groups. In contrast, there was a substantial difference in the proportion of late apoptotic cells. The model group treated with tunicamycin had a late apoptotic cell proportion of 7.54% ± 0.89%, while the control group had an early apoptotic rate of 2.4% ± 0.21%, and the group treated with the highest concentration of TUDCA had an early apoptotic rate of 3.87% ± 1.01% (Fig. 5F to I).

In a chondrocyte model of OA induced by 2 μg/ml tunicamycin, Stress-relaxed HAMA@Lip was prepared to release TUDCA at concentrations of 100, 300, and 500 μM. Coculture experiments showed significant regulation of OA-related genes, including Col II, aggrecan, MMP13, and ADAMTS5, as validated by qPCR analysis. Notably, MMP13 expression decreased steadily with increasing concentrations of HAMA@Lip, whereas ADAMTS5 showed a transient elevation at intermediate doses before declining at higher levels, indicating a differential dose-dependent response.

These findings demonstrate that Stress-relaxed HAMA@Lip effectively alleviates ERS, suppresses apoptosis, and reduces chondrocyte loss, thus providing a promising strategy to delay the progression of OA (Fig. 5J to N).

### In vivo experiments demonstrate that stress-relaxed HAMA@Lip delays OA progression by stress buffering

**Fig. 6.**
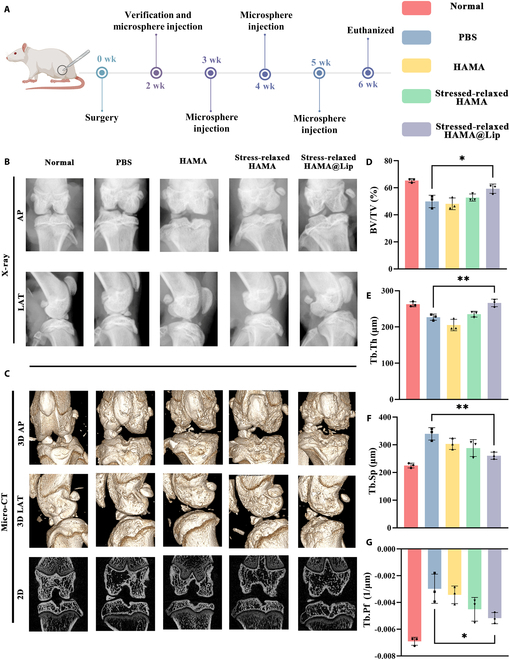
Radiological evaluation of in vivo therapeutic effects of Stressed-relaxed HAMA@Lip. (A) Schematic diagram and timeline of the animal experiment. (B) X-ray images showing the knee joint space and osteophyte formation in both anterior-posterior (AP) and lateral (LAT) views. (C) Micro-CT images illustrating the knee joint and subchondral bone in both 2D and 3D views. (D to G) Quantitative analysis of subchondral bone using bone volume fraction (BV/TV), trabecular thickness (Tb.Th), trabecular separation (Tb.Sp), and trabecular pattern factor (Tb.Pf) (*n* = 3). One-way ANOVA with Tukey’s post hoc test. *P < 0.05, **P < 0.01.

Additional in vivo research confirmed the therapeutic effectiveness of Stress-relaxed HAMA@Lip, referred to as the “cellular shock absorber”, in treating OA. OA in a rat model was induced using the modified Hulth method, which involved anterior cruciate ligament transection, medial collateral ligament transection, and medial meniscus removal (Fig. 6A and Fig. S9). The rats were randomly assigned to 5 groups: sham surgery, PBS, HAMA, Stress-relaxed HAMA, and Stress-relaxed HAMA@Lip.

Intra-articular intervention began on postoperative day 14, at which point no signs of infection, ulcers, or necrosis were observed at the surgical sites in any group. Each group received weekly intra-articular injections of their respective formulations (one injection per week) for a total of 4 consecutive weeks. At the end of week 6 post-surgery, all rats were euthanized, and knee joints were harvested for x-ray imaging, micro-CT, histological analysis, immunohistochemistry, and immunofluorescence staining (Fig. 6B and C).

X-ray imaging revealed that the Stress-relaxed HAMA@Lip group exhibited significantly less osteophyte formation compared to other surgical groups. Although joint space width appeared to increase in all surgical groups compared to the blank group, this observation was attributed to surgical intervention-related joint capsule disruption and transient joint effusion, rather than pathological cartilage degradation. Moreover, the relatively short modeling period (*X* weeks) likely reflects an early-stage OA phenotype, during which characteristic features of advanced OA, such as joint space narrowing or extensive osteophyte formation, are typically not present. Similar findings have been reported in previous studies using modified Hulth or partial meniscectomy models within short observation windows. Therefore, joint space width was not considered a reliable or representative metric of OA progression in this context and was excluded from further quantitative analysis.

Micro-CT scanning provided high-resolution 3D images for precise evaluation of subchondral bone structure and density [59]. The bone volume fraction (BV/TV) was significantly higher in the Stress-relaxed HAMA@Lip group compared to other surgical groups, although slightly lower than the blank group, indicating a reduction in subchondral bone loss during early to mid-stage OA. Trabecular thickness (Tb.Th) increased, trabecular separation (Tb.Sp) decreased, and trabecular pattern factor (Tb.Pf) was reduced in the Stress-relaxed HAMA@Lip group compared to other surgical groups, demonstrating improved trabecular quality and delayed subchondral bone resorption [60] (Fig. 6D to G).

**Fig. 7.**
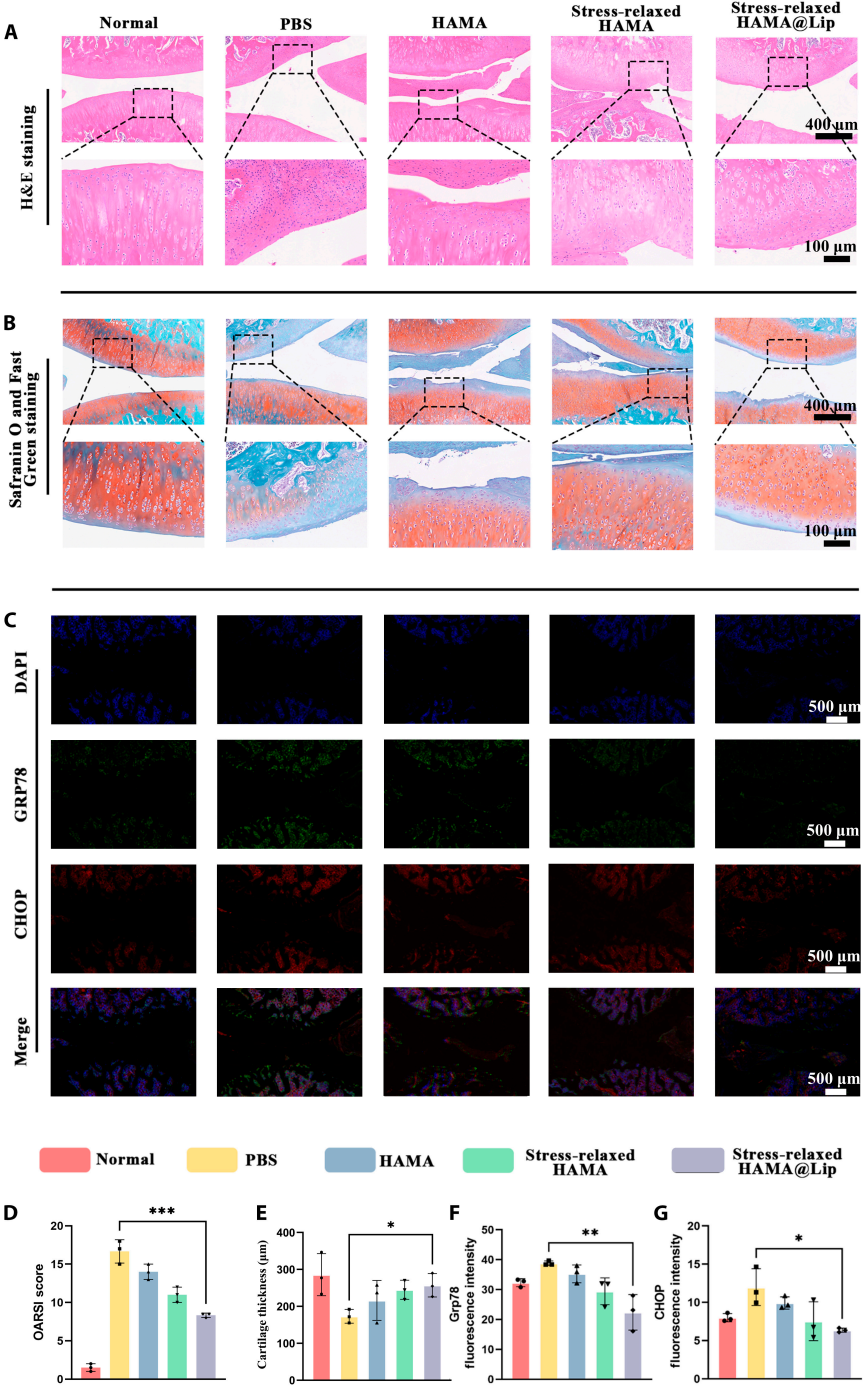
Stressed-relaxed HAMA@Lip alleviates ERS and preserves cartilage structure in OA. (A) Representative H&E staining images of rat knee joints from different groups. (B). Representative Safranin O–Fast Green staining images of rat knee joints from different groups. (C) Representative immunofluorescence images of CHOP and GRP78 in rat joints from different groups. (D) OARSI scoring of OA progression in rats from different groups (*n* = 3); detailed scoring criteria are provided in the Supplementary Materials. (E) Quantitative analysis of articular cartilage thickness in rats from different groups (*n* = 3). (F and G) Quantitative analysis of fluorescence intensity for CHOP and GRP78 in rat joints across different groups (*n* = 3). One-way ANOVA with Tukey’s post hoc test. *P < 0.05, **P < 0.01, ***P < 0.001.

Histological analysis with hematoxylin and eosin (H&E) and Safranin O–Fast Green staining showed typical morphological changes, including cartilage erosion and matrix degradation, in the PBS and HAMA groups (Fig. 7A and B). In contrast, the Stress-relaxed HAMA@Lip group exhibited superior cartilage morphology and structure, with well-aligned chondrocytes and reduced matrix loss. Quantitative analysis using the OARSI (Osteoarthritis Research Society International) scoring system showed that the model group had a score of 16.67 ± 1.50, while the Stress-relaxed HAMA@Lip treatment group had a significantly lower score of 8.33 ± 0.25 (*P* < 0.001), which was better than the HAMA treatment group (14 ± 0.58) and the Stress-relaxed HAMA group (11 ± 1). The thickness of the cartilage also changed with intervention. The model group had a cartilage thickness of 173.16 ± 18.58 μm, while the Stress-relaxed HAMA@Lip treatment group had a significantly higher thickness of 267.11 ± 31.71 μm (*P* < 0.05), which was better than the HAMA treatment group (216.16 ± 54.13 μm) and the Stress-relaxed HAMA group (245.00 ± 25.68 μm). These results collectively demonstrate that the Stress-relaxed HAMA@Lip microsphere system, by continuously releasing TUDCA and providing mechanical support, can significantly reduce cartilage damage and shows superior therapeutic efficacy in the treatment of OA (Fig. 7D and E).

Immunofluorescence staining revealed reduced expression of ERS markers Grp78 and CHOP in the Stress-relaxed HAMA@Lip group compared to other groups. The HAMA microsphere group did not show significant ERS inhibitory effects, while the Stress-relaxed HAMA@Lip treatment group exhibited the strongest ERS regulatory effect. Quantitative analysis of fluorescence intensity revealed that compared with the PBS group, the proportion of CHOP-positive cells in the HAMA@Lip treatment group was reduced by 46.86% (*P* < 0.05), and the proportion of GRP78-positive cells was reduced by 42.29% (*P* < 0.01), which further demonstrating that the synergistic effects of stress buffering and controlled TUDCA release alleviate ERS in cartilage tissue and delay OA progression (Fig. 7C, F, and G).

**Fig. 8.**
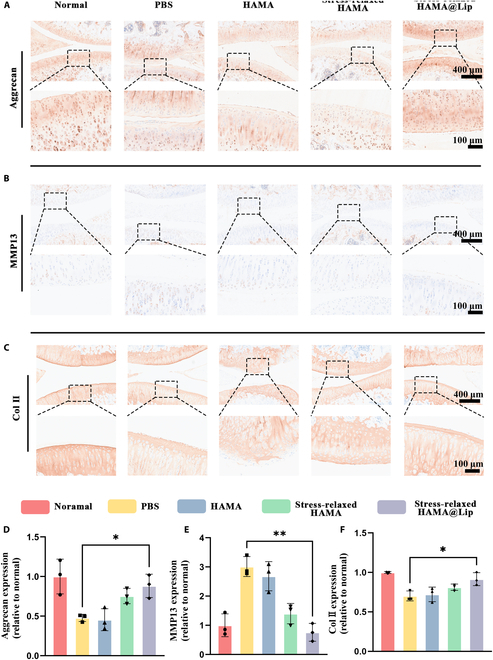
Stressed-relaxed HAMA@Lip inhibits the progression of OA in rats. (A) Representative immunohistochemical staining images of aggrecan in rat joints from different groups. (B) Representative immunohistochemical staining images of MMP13 in rat joints from different groups. (C) Representative immunohistochemical staining images of Col II in rat joints from different groups. (D) Quantitative analysis of aggrecan expression in rat joints normalized to the normal group (*n* = 3). (E) Quantitative analysis of MMP13 expression in rat joints normalized to the normal group (*n* = 3). (F) Quantitative analysis of Col II expression in rat joints normalized to the normal group (*n* = 3). One-way ANOVA with Tukey’s post hoc test. *P < 0.05, **P < 0.01.

Immunohistochemistry results demonstrated that the Stress-relaxed HAMA@Lip group had significantly higher expression levels of Col II and aggrecan and lower levels of MMP13 compared to the PBS and HAMA groups (Fig. 8A to C). The experimental results showed that compared with the blank control group, the expression of aggrecan and Col II in the OA model group was significantly reduced (by 55% and 32%, respectively; *P* < 0.05), while the expression of MMP13 increased by 3.3 times (*P* < 0.01), indicating significant degradation of the cartilage matrix in the model group. In the HAMA microsphere treatment group, there was little change in the expression of aggrecan and Col II, and the decrease in MMP13 expression was not statistically significant. Notably, the Stress-relaxed HAMA@Lip group provided the greatest protection against OA-related damage. The expression of aggrecan and Col II in this group was restored to 82% and 88% of the control group, respectively (*P* < 0.05). Meanwhile, the expression of MMP13 was further reduced to 18% of that in the model group (*P* < 0.01), even lower than that in the blank control group (Fig. 8D to F).

## Discussion

This study delves deeper into the critical role of the intra-articular mechanical environment in the progression of OA. Abnormal mechanical vibrations and stress not only are primary physical factors causing OA but also activate downstream biological signaling pathways in response to mechanical stimuli. Beyond the impact on ERS, the mechanical environment exacerbates OA progression through the activation of multiple biological pathways. Known pathways include the calcium signaling pathway (Piezo1/2, TRPV4, CaMKII, calcineurin), integrin-mediated signaling (integrins, FAK, Src family kinases), and the Hippo signaling pathway (YAP/TAZ, MST1/2, LATS1/2). These pathways may serve as potential therapeutic targets for mechanically induced diseases, and modulating their signaling cascades could provide novel strategies for addressing such conditions.

Hydrogel material modification is a well-established approach in materials science. In earlier studies, we functionalized HA with MA to introduce photopolymerization capabilities in the presence of photoinitiators. In this study, HAMA was further oxidized and aminated, enabling Schiff base reactions between reactive amine and aldehyde groups, which allowed fine-tuning of hydrogel mechanical properties. Other modification strategies, such as incorporating reinforcing phases, designing fibrous structures, or creating multiphase structures, also effectively enhance hydrogel performance. Our approach demonstrates high tunability, self-healing capacity, and dynamic responsiveness, coupled with rapid photopolymerization and good controllability. However, challenges remain, including complex fabrication processes and material heterogeneity, necessitating further optimization. Nevertheless, these strategies provide innovative design ideas for materials tailored to diverse mechanical requirements. The dynamic reversibility of imine bonds plays a critical role in enhancing the energy dissipation capacity of hydrogels compared to traditional covalently crosslinked networks such as methacrylated systems [61,62]. Under mechanical stress, the breakage and reformation of imine bonds allow for temporary stress redistribution, effectively reducing peak stress concentrations—a mechanism that closely mimics the viscoelastic stress buffering behavior of native cartilage, which relies on the dynamic dissociation of noncovalent interactions within the collagen–proteoglycan network. Studies have shown that imine-bonded hydrogels exhibit significantly higher loss modulus (*G*″) than purely covalent networks, confirming their superior energy dissipation at the molecular level.

Moreover, the dynamic nature of imine bonds enables a delicate balance between crosslinking density (stiffness) and stress relaxation rate. While higher crosslinking density enhances initial modulus, it restricts chain mobility and slows stress relaxation. By optimizing the oxidation/amination ratio, the exchange kinetics of imine bonds can be tuned to achieve rapid energy dissipation without compromising structural integrity. In recent years, significant progress has been made in the regulation of hydrogel mechanical properties. Numerous studies have demonstrated that by optimizing crosslinking chemistry and network architecture, researchers can independently achieve either high mechanical strength or rapid stress relaxation. For instance, one study reported a novel rigid and self-healing hydrogel that achieved a high modulus (50 MPa) and tensile strength (4.2 MPa) through polymer entanglement within coplanar nanoconfinement [63]. Conversely, another study designed a low-crosslinking-density imine network, where stress relaxation properties were adjusted by varying concentrations, but the modulus remained limited to 50 to 80 Pa [64]. The future challenge lies in integrating the advantages of both approaches—i.e., maintaining a high modulus while enabling rapid stress relaxation through precise control of dynamic bond exchange kinetics (e.g., pH-/temperature-responsive imine bonds or metal coordination bonds). Recently proposed “dual dynamic network” designs (where covalent crosslinks ensure strength, while reversible crosslinks regulate relaxation) offer a promising pathway toward this goal. However, further optimization of long-term stability and fatigue resistance remains necessary to meet the demands of load-bearing tissue engineering applications.

In this study, we successfully developed pressure-relaxation hydrogel microspheres that mimic the buffering function of cartilage matrix and act as cellular shock absorbers to slow OA progression. By integrating stress relaxation properties of articular cartilage with the sustained-release mechanism of TUDCA, these microspheres effectively reduced ERS in chondrocytes and significantly alleviated mechanical stress. However, despite their efficacy in mitigating ERS and delaying OA progression, certain limitations persist. Due to the difficulty in obtaining healthy human cartilage samples for mechanical testing, we referred to previously reported stress relaxation parameters of human cartilage in the literature as a benchmark. Although this indirect comparison may introduce some variability, it provides a reasonable and relevant reference point for evaluating the viscoelastic design of our microspheres.

Our strategy does not provide sufficient cartilage regeneration signals to reverse the pathological progression of OA. Additionally, the pressure-relaxation hydrogel microspheres require periodic replenishment to maintain elastic stress relief in joints. Future efforts will explore the integration of cartilage regeneration bioengineering approaches. Such advancements could not only sustain stress relief but also accelerate cartilage regeneration, offering a novel therapeutic avenue for OA treatment.

## Conclusion

In this study, we successfully developed a pressure-relaxation hydrogel microsphere, named “Stress-relaxed HAMA@Lip”, designed to buffer mechanical stress and improve the joint mechanical environment, thereby delaying OA progression. By integrating multichannel microfluidic technology with cationic liposome preparation techniques, we achieved efficient encapsulation and sustained release of TUDCA. These hydrogel microspheres effectively alleviated mechanical stress on articular cartilage during movement, reduced ERS in chondrocytes, and decreased chondrocyte apoptosis. The experimental results demonstrated that Stress-relaxed HAMA@Lip significantly mitigated cartilage degeneration, preserved cartilage structural integrity, and delayed OA progression in a rat model. The sustained release of TUDCA inhibited the expression of ERS markers and enhanced chondrocyte viability. In conclusion, this study introduces a novel and effective strategy for OA treatment, emphasizing the potential of hydrogel microspheres in reducing mechanical stress and alleviating ERS. These findings lay a theoretical foundation for the development of biomaterial-based therapies for OA and present an innovative therapeutic approach for clinical applications.

## Materials and Methods

### Materials

WYRGRL (Trp-Tyr-Arg-Gly-Arg-Leu), DSPE-PEG_2000_-NH_2_, and DSPE-PEG_2000_-WYRGRL were provided from China Apeptide Co. Ltd (Shanghai, China). Cholesterol, octadecylamine, lecithin, ThT, sodium periodate, oxalyl dihydrazide, 1-(3-dimethylaminopropyl)-3-ethylcarbodiimide hydrochloride (EDC), and 1-hydroxybenzotriazole (HOBt) were provided by Aladdin Industrial Co. Ltd. (Shanghai, China). TUDCA was provided by Yeasen Biotechnology Co. Ltd. (Shanghai). HA (molecular weight = 168 kDa), MA (≥94%), and lithium phenyl(2,4,6-trimethylbenzoyl) phosphinate (LAP, ≥95%) were provided by Sigma-Aldrich. Dulbecco’s modified Eagle’s medium/Nutrient Mixture F-12 (DMEM/F12), 0.25% trypsin-EDTA, fetal bovine serum (FBS), and antibiotics were purchased from Invitrogen (CA, USA). Tunicamycin (SC0393) was purchased by Beyotime Biotechnology. Rhodamine phalloidin and 4′,6-diamidino-2-phenylindole (DAPI) dihydrochloride were purchased by MedChemExpress.

Antibodies were used at a dilution of 1:1,000 for Western blot analysis and 1:200 for immunofluorescence, unless otherwise specified. Col II antibody (AF0135), MMP13 antibody (AF5355), aggrecan antibody (DF7561), ADAMTS5 antibody (DF13268), and β-actin antibody (AF7018) were purchased by Affinity Biosciences. ATF-6 (D4Z8V) antibody (#65880), CHOP (L63F7) antibody (#2895), BiP (Grp78) antibody (#3183), and phospho-eIF2α (Ser^51^) antibody (#9721) were purchased by Cell Signaling Technology.

RNA sequences for qPCR are given in the Supplementary Materials. All RNAs are purchased by Sangon Biotech (Shanghai) Co. Ltd.

### Synthesis and characterization of cationic liposomes

WYRGRL was conjugated to DSPE-PEG_2000_-NHS via a Mannich reaction to synthesize DSPE-PEG_2000_-WYRGRL. Briefly, 100 mg of DSPE-PEG_2000_-NHS was dissolved in DMF (*N*,*N*-dimethylformamide), and WYRGRL peptide (1.1 equivalents), along with triethylamine (3.0 equivalents), was added and fully dissolved. The mixture was then allowed to react for 12 h. The reaction mixture was placed in dialysis bag with a molecular weight cutoff of 1,000 Da and dialyzed against pure water for 24 h. The dialysate was collected, freeze-dried to obtain the product, and stored for subsequent experiments. ^1^H NMR and mass spectrometry were used to validate the successful synthesis of DSPE-PEG_2000_-WYRGRL. The synthetic yield of DSPE-PEG_2000_-WYRGRL was measured by HPLC.

Cationic liposomes Lipo-Wyrgrl@TUDCA were prepared using the reverse evaporation method. Phospholipids, cholesterol, stearylamine, DSPE-PEG_2000_-WYRGRL, DSPE-PEG_2000_-NH_2_, and TUDCA were dissolved in a mixed solution of dichloromethane (HPLC grade, ≥99.9%) and water (volume ratio of 3:1) at a mass ratio of 5:1:3:1.2:1.5:1. The mixture was sonicated to form a stable oil-in-water emulsion and then transferred to a 250-ml flask. Once the organic solvent was eliminated through vacuum rotary evaporation at 60 °C, a clear and uniform lipid film was produced. This film was then hydrated in PBS with a pH of 7.4 at 37 °C. The resulting liposomes were subjected to probe sonication (30% amplitude, 5 min) and extruded through a filter to yield the Lipo-Wyrgrl@TUDCA cationic liposomes.

The particle size and zeta potential of Lipo-Wyrgrl@TUDCA were both determined using a Malvern laser particle size analyzer (DLS). The morphological characteristics of Lipo-Wyrgrl@TUDCA were observed by TEM. For observation, Lipo-Wyrgrl@TUDCA was diluted and allowed to form a monolayer or low-density distribution on a TEM grid. Phosphotungstic acid (PTA) was applied as a staining agent, and after drying, the samples were examined using a TEM (FEI Tecnai F20).

### Synthesis and characterization of stress-relaxed HAMA

The synthesis of HAMA has been previously reported in detail. In brief, 10 g of HA (molecular weight = 168 kDa) was reacted with 2 ml of MA overnight under alkaline conditions. After centrifugation to remove impurities, the product was purified by dialysis for 4 d, followed by freeze-drying to obtain HAMA, which was stored at −20 °C for further use.

HAMA was oxidized to obtain HAMA-CHO. HAMA (0.8 g) was dissolved in 50 ml of deionized water, and 450 mg of sodium periodate (dissolved in 10 ml of water) was added to make the final concentration of sodium periodate 40 mM. The reaction was carried out at room temperature (20 °C) in the dark for 4 h under constant stirring. The product was then dialyzed against deionized water using a membrane with a molecular weight cutoff of 3.5 kDa. The dialysis solution was replaced every 6 h during the first day and every 12 h thereafter for a total of 3 d. Finally, the purified HAMA-CHO was freeze-dried and stored at −20 °C until further use. For the synthesis of HAMA-NH_2_, 2 g of HAMA was dissolved in 100 ml of 0.1 M MES [2-(*N*-morpholino)ethanesulfonic acid] buffer (pH 6.0). EDC (1.2 g) and HOBt (0.8 g) were added to achieve a final concentration of 60 mM for both reagents, and the mixture was pre-activated for 30 min at 4 °C. Subsequently, adipic acid dihydrazide (4.58 g) was added to give a final concentration of 260 mM, and the reaction was allowed to proceed for 12 h at 4 °C under constant stirring. The product was dialyzed against a 25 g/l sodium chloride solution using the same dialysis protocol as described above. The final product, HAMA-NH_2_, was freeze-dried and stored at −20 °C. The successful synthesis of HAMA, HAMA-CHO, and HAMA-NH_2_ can be verified, and their degree of substitution was calculated through ^1^H NMR detection.

Rheological tests were conducted using a Discovery Hybrid Rheometer (TA Instruments) equipped with a 20-mm parallel plate geometry and a gap size of 500 μm. HAMA, HAMA-CHO, and HAMA-NH_2_ were prepared as 2 wt % solutions. Lipo-Wyrgrl@TUDCA, previously prepared, was mixed with the materials at the required volume ratios. The mixtures were rapidly combined, and 0.5% LAP was added as a photoinitiator. After loading the samples onto the rheometer, a 2-min equilibration period was allowed to ensure temperature stability. The rheometer was maintained at 37 °C throughout the tests. Crosslinking was achieved by irradiating the samples with a 405-nm blue light curing device (30% intensity) for 300 s. Frequency sweeps (5% strain, 0.1 to 10 Hz) and amplitude sweeps (1 Hz, 1% to 200% strain) were performed to measure the elastic and viscous module. Stress relaxation characteristics were evaluated by applying a 5% strain for 300 s. Each experiment was repeated 3 times to ensure reproducibility.

Next, stress-relaxed HAMA@Lip was prepared using a 3-phase microfluidic technique. The aqueous phase was divided into an inner and an outer phase; the inner phase consisted of 2 wt % HAMA-CHO and 0.5 wt % LAP, while the outer phase was composed of Lipo-Wyrgrl@TUDCA, 2 wt % HAMA-NH_2_, and 0.5 wt % LAP. The oil phase consisted of paraffin oil combined with 8 wt % Span 80. The microdroplets were subsequently cooled to −40 °C and underwent crosslinking via UV light exposure. Following this, the paraffin oil and other additives were removed, yielding the stress-relaxed HAMA@Lip.

Characterization of the microspheres involved bright-field microscopy (LSM800) to check hydrogel synthesis and particle size, LSCM (laser scanning confocal microscopy) (Zeiss) to observe DIR dye-loaded liposome distribution, and UV spectrophotometry (Eppendorf) to measure TUDCA encapsulation and release. Efficiency was calculated as *W*/*W*_0_ × 100%, with release studied over 25 d in PBS (pH 7.4). Scanning electron microscopy (SEM, Zeiss) was used to examine the morphology, porosity, and size of the samples after freeze-drying.

### Isolation and culture of primary rat chondrocytes

To obtain primary chondrocytes, we isolated cells from the articular cartilage of 1-week-old rats. Initially, the cartilage tissue was physically minced and digested overnight at 37 °C with 0.2% collagenase II to release the cells. After digestion, the cell suspension was filtered through a 70-μm cell strainer to remove undigested tissue debris and washed twice with F12/DMEM medium containing 10% FBS to terminate the digestion process. The purified cells were subsequently plated in culture dishes and maintained in DMEM/F12 medium that was enriched with 5% FBS and 1% penicillin/streptomycin. The cells were cultured at 37 °C under an atmosphere containing 5% CO_2_. Cell passaging was carried out once the cells achieved 80% to 90% confluence. To preserve the primary characteristics of the cells, all experiments were conducted using cells from the third to the fifth passage.

### Biocompatibility testing and the establishment of in vitro models

The live/dead staining assay and the CCK-8 assay were employed to assess the impact of Stress-relaxed HAMA@Lip (2.5 mg/ml) on primary chondrocyte proliferation. Cells (1.0 × 10^4^ ml^−1^) were placed in the lower chamber of a 24-well transwell plate (Corning), with Stress-relaxed HAMA@Lip (2.5 mg/ml) in the upper chamber. On days 1, 2, and 3, cells were stained with 250 μl of calcein-AM/PI solution (Beyotime) for 30 min and viewed under a fluorescence microscope. Cell proliferation was also measured using a CCK-8 kit (Beyotime). Additionally, the CCK-8 assay was used to determine the toxicity of various actinomycin concentrations on primary chondrocytes. Primary chondrocytes (2 × 10^4^) were seeded in 6-well plates, treated with different actinomycin concentrations once they reached 70% confluence, and incubated for 24 h to establish the model. Cell survival was then observed using the CCK-8 kit.

### Western blot

Cells were rinsed twice with ice-cold PBS and lysed using radioimmunoprecipitation assay buffer containing protease and phosphatase inhibitors. The lysates were incubated on ice for 10 min and then centrifuged to remove debris. The resulting supernatants were subjected to sodium dodecyl sulfate–polyacrylamide gel electrophoresis and transferred to nitrocellulose membranes. These membranes were blocked with a rapid blocking solution and incubated with primary antibodies in 3% bovine serum albumin (BSA) in TBST (tris-buffered saline with Tween 20). Subsequently, they were incubated with either anti-rabbit horseradish peroxidase (HRP)-conjugated immunoglobulin G (IgG) (1:4,000) or anti-mouse HRP-conjugated IgG (1:3,000), and the signals were detected using enhanced chemiluminescence (ECL) reagent. The grayscale intensity of each band was quantified using ImageJ software, and target protein expression was normalized to β-actin levels.

### Immunofluorescence staining

Chondrocytes were seeded into a confocal 24-well plate and cultured in DMEM/F12 medium containing 5% FBS. Subsequently, the cells were washed twice with PBS (25 °C) and fixed with 4% paraformaldehyde at room temperature for 10 min. After that, the cells were permeabilized in PBS containing 0.2% Triton X-100 for 10 min and blocked with PBS containing 5% BSA for 30 min. Then, the cells were incubated with the primary antibody overnight at 4 °C. The following day, the cells were washed with PBS and incubated with the corresponding secondary antibody for 30 min at room temperature. The cytoskeleton was stained with rhodamine-labeled phalloidin, and after washing with PBS, the nuclei were stained with DAPI. The stained cells were observed and recorded under a confocal microscope.

### Quantitative PCR

Real-time qPCR was used to evaluate the impact of Stress-relaxed HAMA@Lip on OA after regulating ERS. Primary chondrocytes from rats were first treated with actinomycin for 24 h and then cocultured with Stress-relaxed HAMA@Lip loaded with different concentrations of TUDCA for an additional 24 h. RNA was extracted from the cultured cells, and cDNA was synthesized using the RevertAid First Strand cDNA Synthesis Kit. qPCR was conducted with FastStart Universal SYBR Green Master. Primer sequences for aggrecan, ADAMTS-5, MMP13, Col II, and actin are provided in Table S1. Relative gene expression levels were calculated using the 2^−ΔΔCT^ method, and all experiments were performed in triplicate.

### Rat model of OA

The animal study was approved by the Research Ethics Committee of Ruijin Hospital (IACUC-20240301-01). Thirty male Sprague-Dawley rats (6 to 8 weeks old, ~200 g) from Vital River Laboratory were used. For the OA model, rats were anesthetized, and the MCL (medial collateral ligament), ACL (anterior cruciate ligament), and medial meniscus were cut, and then the joint was sutured. The sham group (*n* = 6) only received anesthesia and skin incision. The OA rats (*n* = 24) were divided into 4 groups, with model effectiveness confirmed at 4 weeks. From weeks 5 to 8, 100 μl of PBS, HAMA, Stress-relaxed HAMA, or Stress-relaxed HAMA@Lip was injected into the knee joints of each group.

### X-ray and micro-CT

At 5 weeks post-surgery, x-ray imaging was performed on all rats using an American Faxitron X-ray system (32 kV). Lateral and anteroposterior x-ray images of the right knee joint were captured. Following this, all rats were euthanized to collect knee joint samples, which were then analyzed using a high-resolution micro-CT imaging system (SkyScan1172) for arthrography. Based on the micro-CT scan results and 3D reconstruction, the formation of osteophytes and the condition of the subchondral bone were evaluated.

### Histological analysis

The knee joints were fixed in 4% paraformaldehyde, decalcified for 45 d, and embedded in paraffin for histological and immunohistochemical evaluations. The samples were then sectioned, with coronal sections stained using H&E and Safranin O–Fast Green for histological analysis. The OARSI scoring system was applied to assess the pathological condition of the knee joints. Sections were incubated overnight at 4 °C with rabbit polyclonal antibodies against Col II, aggrecan, and MMP13, followed by a 1-h secondary antibody treatment, and stained with 3,3′-diaminobenzidine (DAB) substrate. Protein expression was quantified using ImageJ software based on positive staining area and intensity. For immunofluorescence staining, sections were incubated overnight with CHOP and GRP78 antibodies and then treated with fluorescein isothiocyanate or Cy5 methyltetrazine at 4 °C for 30 min, followed by a 5-min DAPI incubation, and imaged using fluorescence scanning. Fluorescence signal intensity was analyzed using ImageJ to evaluate relative protein expression across groups.

### Statistics analysis

Statistical analysis was conducted using GraphPad Prism 9. Comparisons of experimental data between 2 groups were performed using the Student’s *t* test. One-way or 2-way analysis of variance (ANOVA) was used to compare experimental data among multiple groups. *P* < 0.05 was considered to indicate a statistically significant difference.

## Data Availability

The data that support the findings of this study are available from the corresponding author upon reasonable request.
