# Supplementary material for: Cell Shock Absorption via Stress Relaxation Hydrogel Microspheres for Alleviating Endoplasmic Reticulum Stress in Chondrocytes
Source: Research (Wash D C). 2025 Jul 17;8:0777. doi: 10.34133/research.0777 (PMC12267986; doi:10.34133/research.0777)
Supplement: Supplementary 1 — Figs. S1 to S9 Tables S1 and S2 [file research.0777.f1.docx]

**Supplementary Figures**

**Fig. S1.** HPLC of DSPE-PEG_2000_-Wyrgrl

**Fig. S2.**  Mass Spectrometry of DSPE-PEG^2000^-Wyrgrl


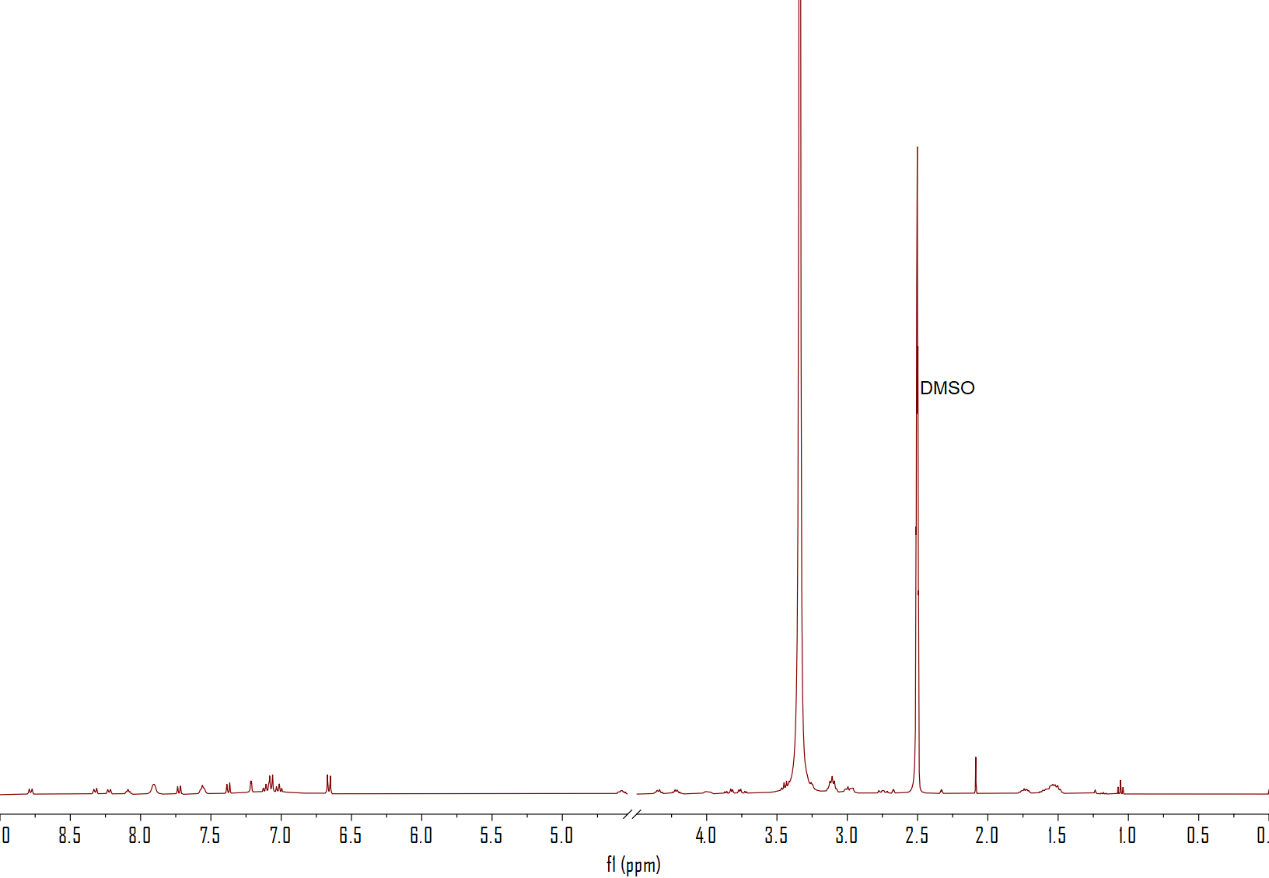


**Fig.S3.** HNMR of DSPE-PEG^2000^-Wyrgrl


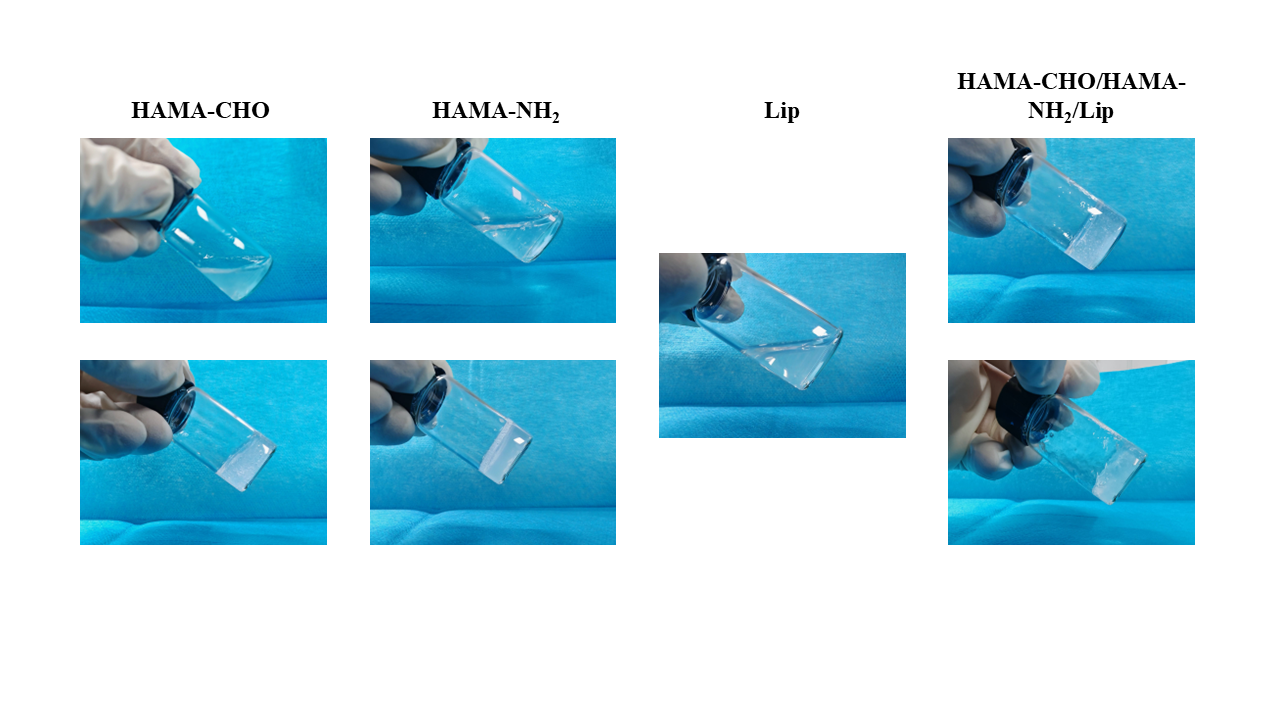


**Fig.S4.** Synthesized HAMA-CHO, HAMA-NH_2_,Lip and their mixtures before UV irradiation and after 3 min of UV irradiation


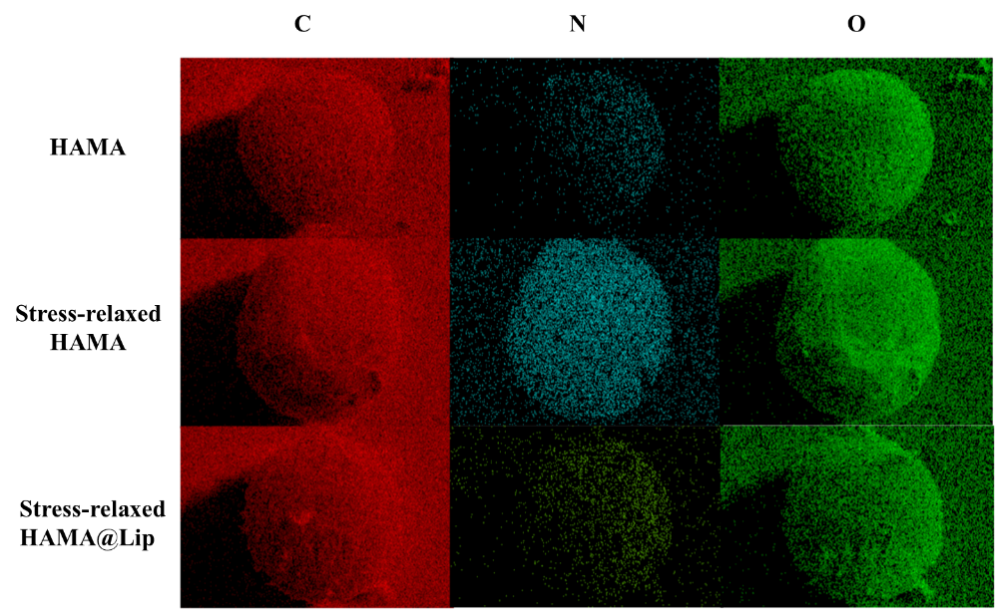


**Fig.S5.** Elemental mapping of different hydrogel microspheres.


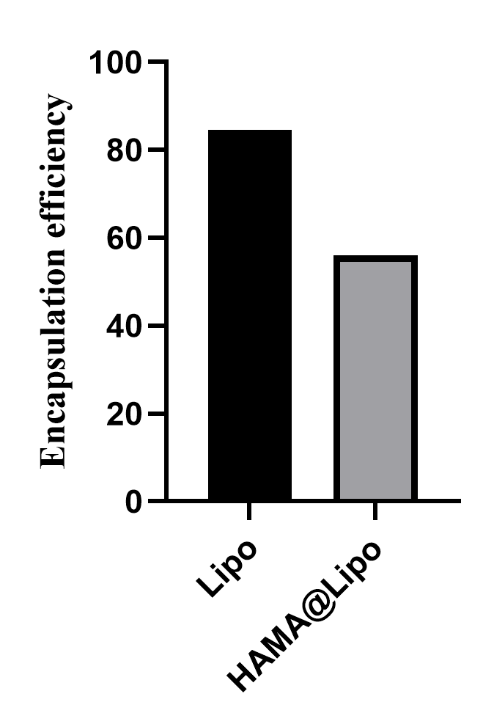


**Fig.S6.** Encapsulation rate of Lip and stress-relaxed HAMA@Lip.


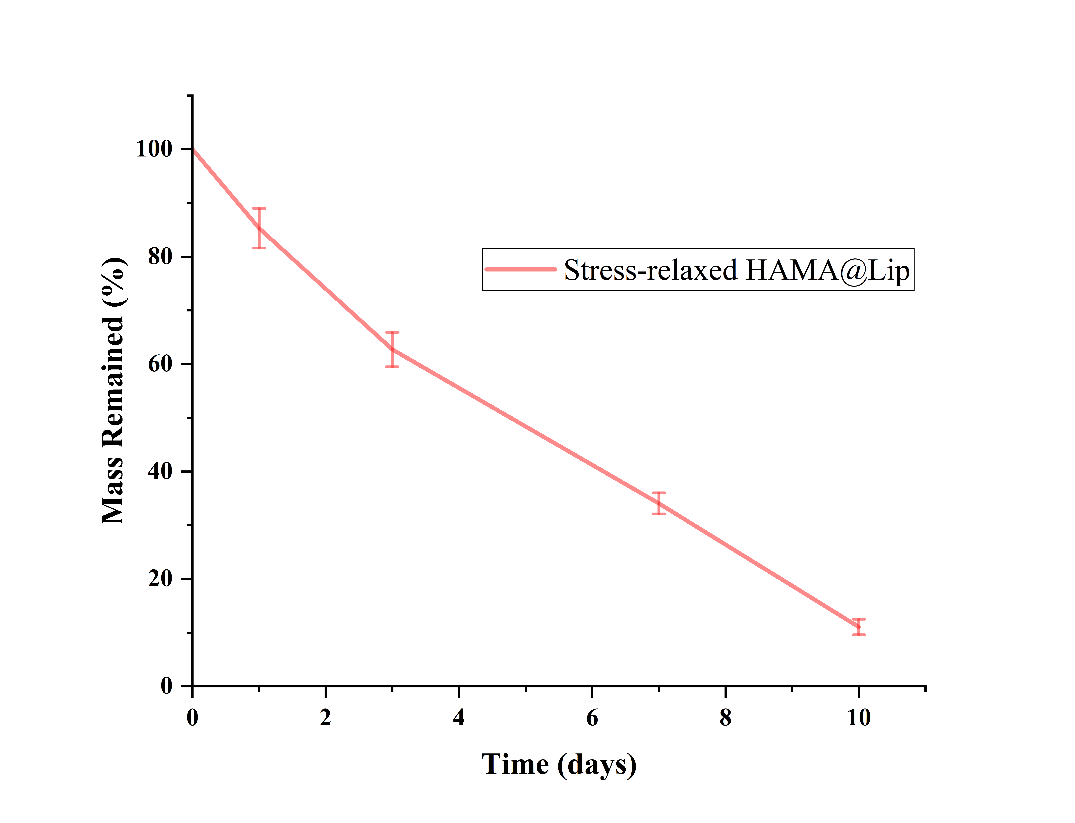


**Fig.S7.** Degradation rate of stress-relaxed HAMA@Lip.


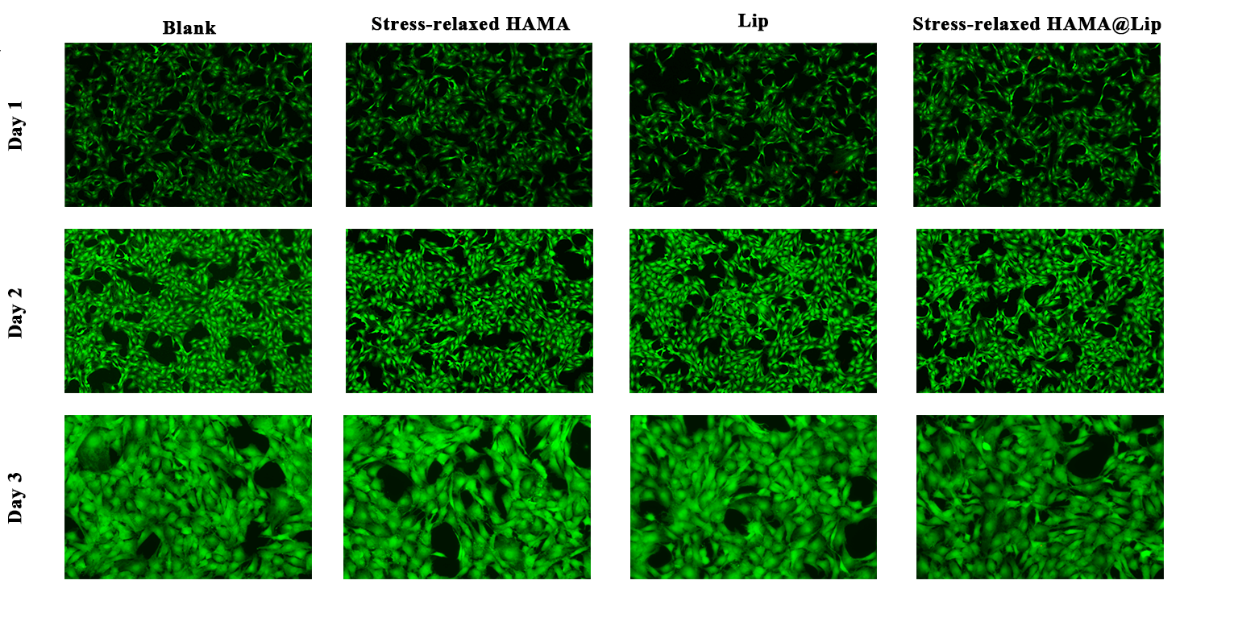


**Fig.S8.** Biocompatibility of different groups of materials, live-dead staining of co-cultures on days 1, 2 and 3.


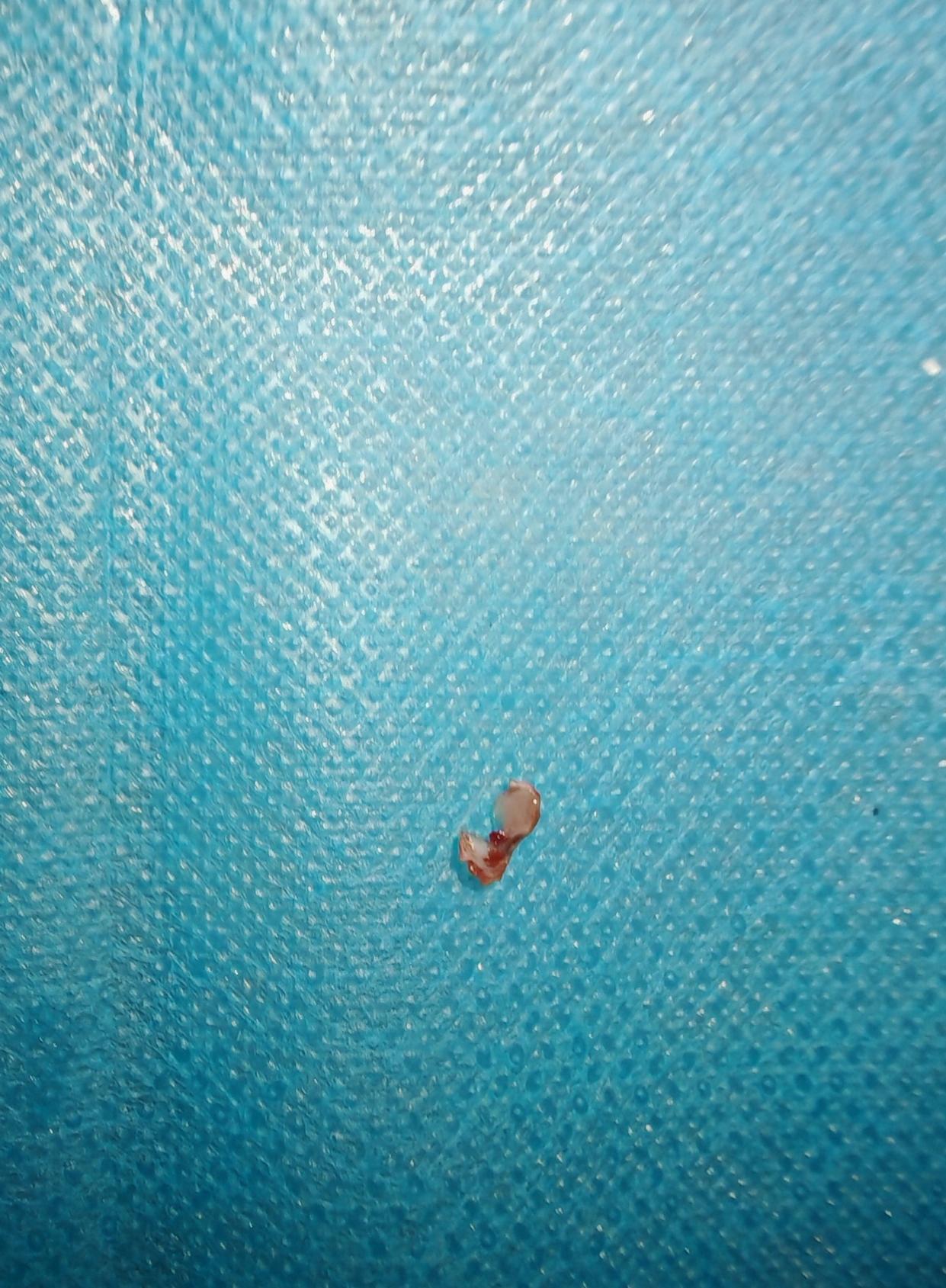

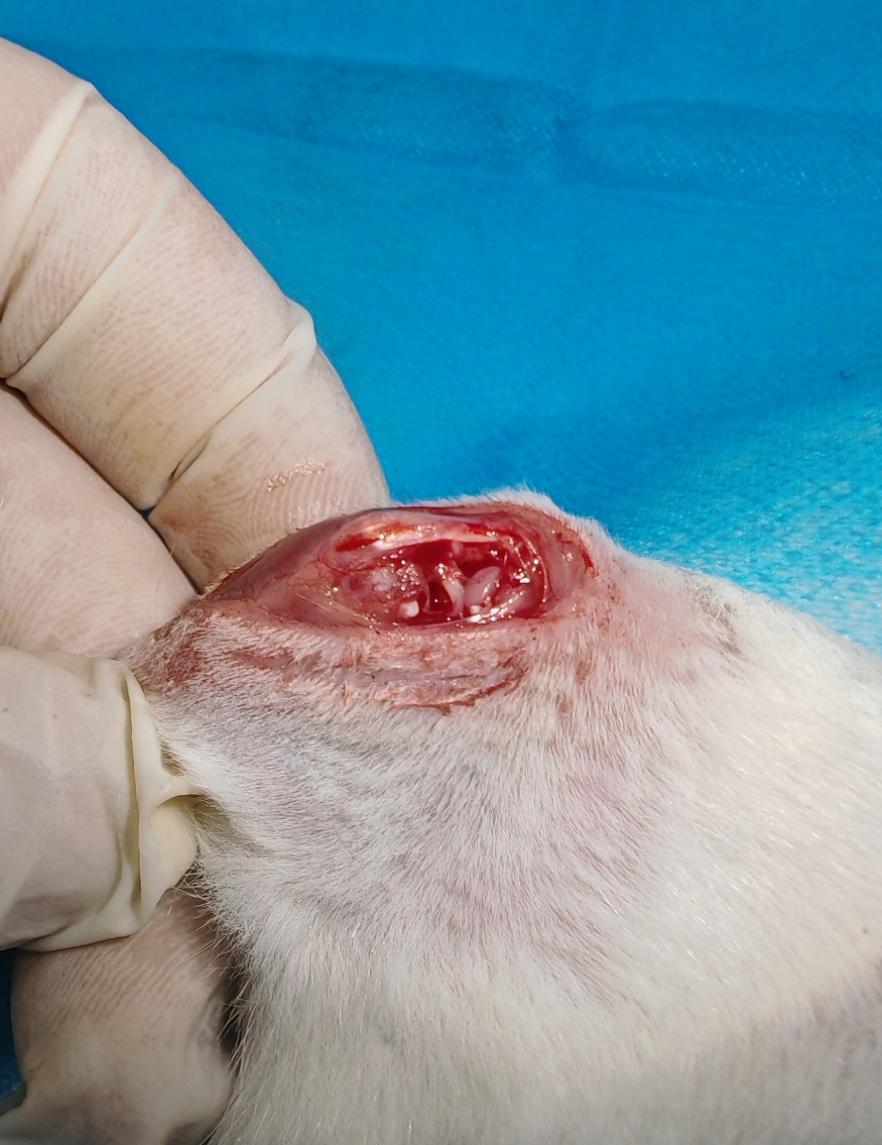


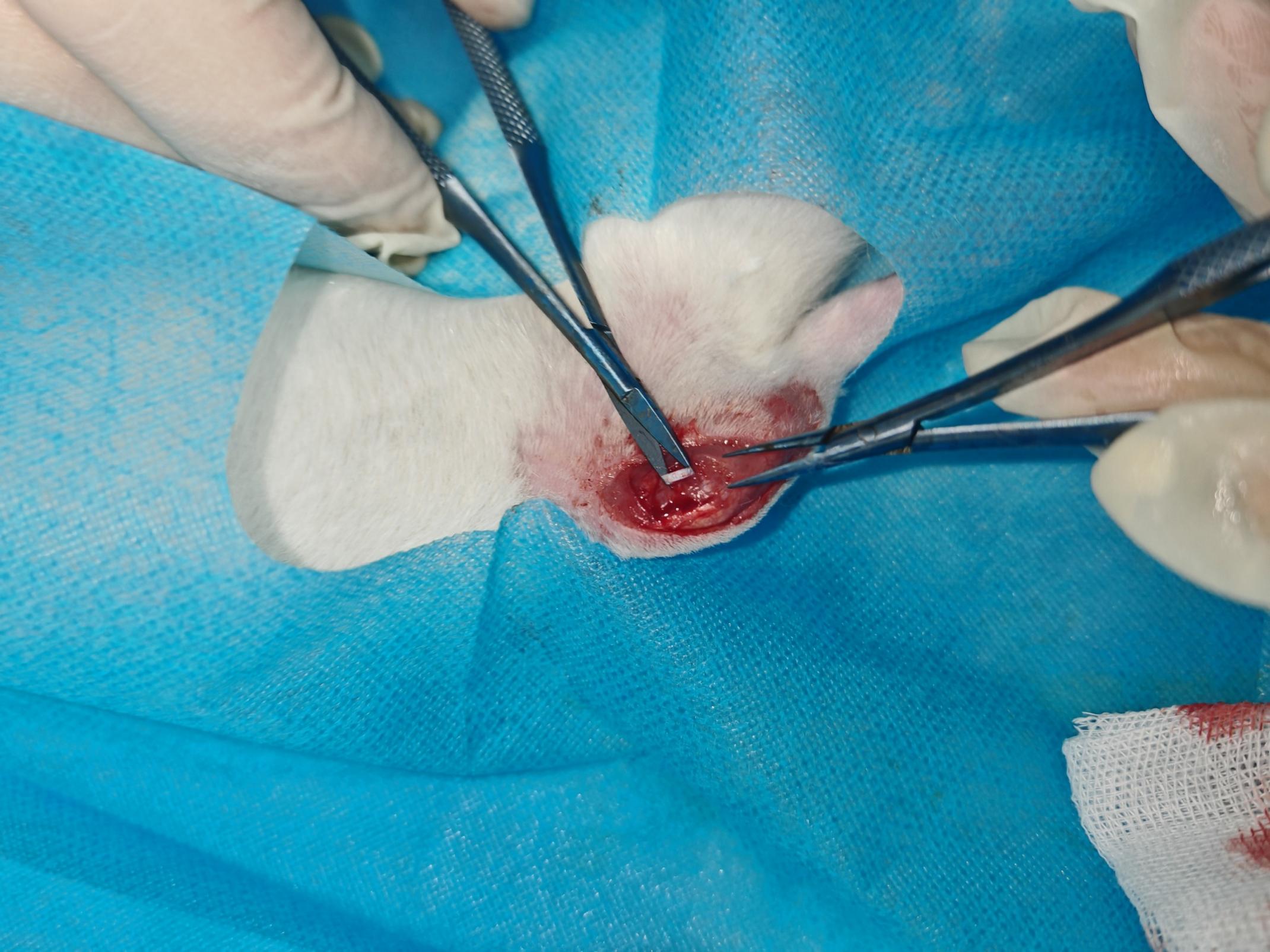

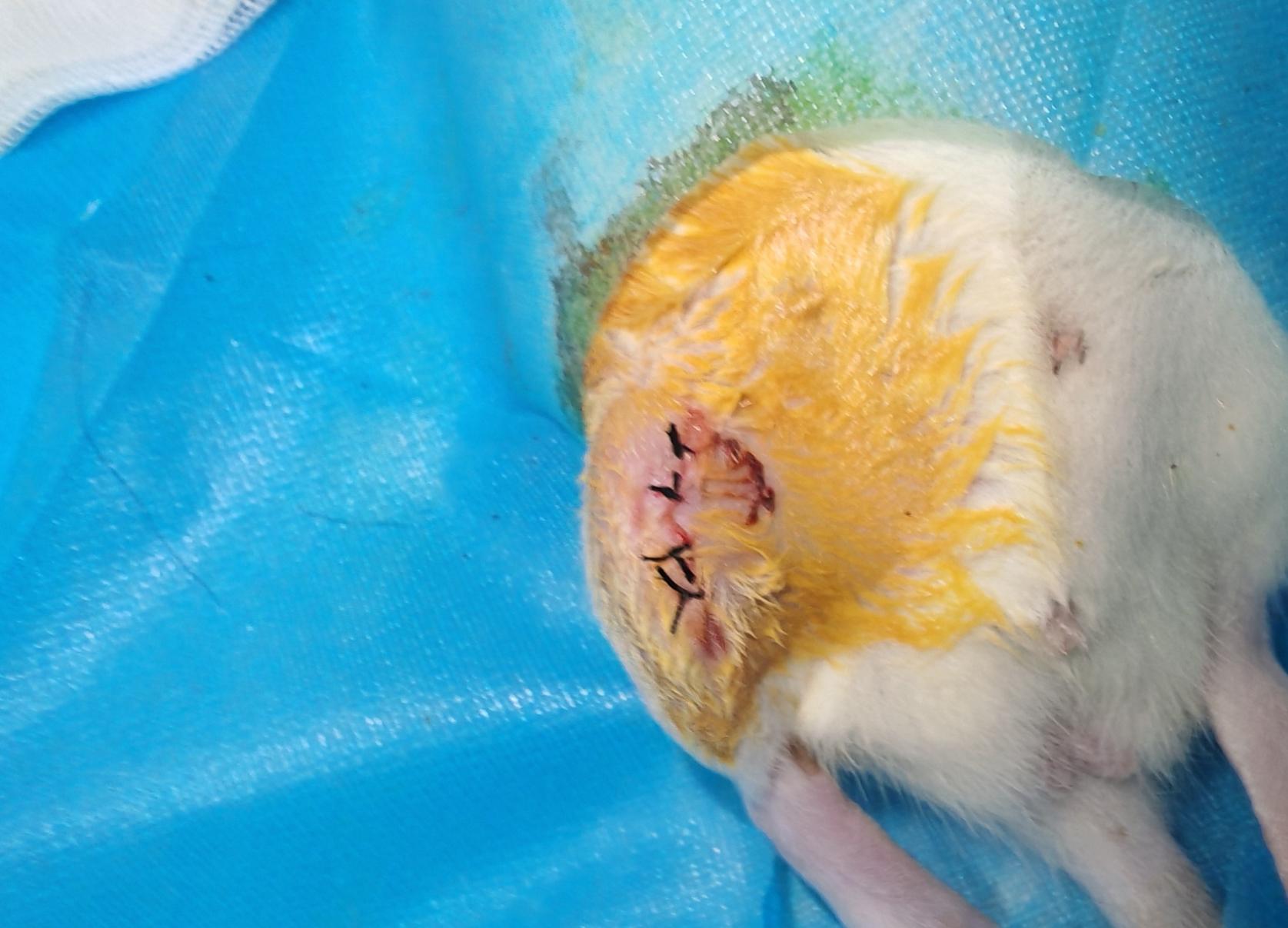


**Fig.S9.** Modified Hulth's method of modeling the process of osteoarthritis in rats, with the meniscus, the exposed anterior cruciate ligament, the lateral tibial collateral ligament and the sutured wound shown in sequence

**Table S1.** Primer sequences for real-time PCR studies

| **Gene** | **Forward** | **Reverse** |
| --- | --- | --- |
| **Actin** | CCCGCGAGTACAACCTTCTT | CGCAGCGATATCGTCATCCA |
| **ADAMTS5** | CCCAAATACGCAGGTGTCCT | ACACACGGAGTTGCTGTAGG |
| **MMP-13** | CTGGGCCCTGAATGGGTATG | CTCAAAGTGAACCGCAGCAC |
| **Aggrecan** | GGGACCTGTGTGAGATCGAC | GGTCGGGAAAGTGGCGATAA |
| **Col2** | ATCGCCACGGTCCTACAATG | CATCGCAGAGGACATTCCCA |

**Table S2.** **The OARSI criteria.**

Cartilage degeneration score

| Parameter | Grade | Description |
| --- | --- | --- |
| Cartilage  degeneration | 0 | No degeneration |
|  | 1 | Minimal degeneration; 5-10% of the total projected cartilage area affected by matrix or chondrocyte loss |
|  | 2 | Mild degeneration; 11-25% affected |
|  | 3 | Moderate degeneration; 26-50% affected |
|  | 4 | Marked degeneration; 51-75% affected |
|  | 5 | Severe degeneration; greater than 75% affected |
